# 9-(4-Hy­droxy-3-meth­oxy­phen­yl)-3,3,6,6-tetra­methyl-3,4,5,6-tetra­hydro-9*H*-xanthene-1,8(2*H*,7*H*)-dione

**DOI:** 10.1107/S1600536810020258

**Published:** 2010-06-05

**Authors:** Noorhafizah Hasanudin, Aisyah Saad Abdul Rahim, Nornisah Mohamed, Ching Kheng Quah, Hoong-Kun Fun

**Affiliations:** a School of Pharmaceutical Sciences, Universiti Sains Malaysia, 11800 USM, Penang, Malaysia; b X-ray Crystallography Unit, School of Physics, Universiti Sains Malaysia, 11800 USM, Penang, Malaysia

## Abstract

In the title compound, C_24_H_28_O_5_, the two cyclo­hexene rings adopt envelope conformations, and the planes through the coplanar atoms makes dihedral angles of 82.86 (6) and 77.90 (6)° with the benzene ring. The two cyclo­hexene rings make a dihedral angle of 5.33 (6)° between their least-squares planes. The pyran ring adopts a flattened boat conformation. In the crystal packing, mol­ecules are linked into two-dimensional networks parallel to the *ab* plane *via* O—H⋯O and C—H⋯O inter­actions.

## Related literature

For the synthesis of the title compound, see: Venkatesan *et al.* (2008 ▶). For general background to and the biological activity of xanthene derivatives, see: Hafez *et al.* (2008 ▶); Ashry *et al.* (2006 ▶); Sill & Sweet (1977 ▶); Ion (1997 ▶); Chibale *et al.* (2003 ▶). For reference bond lengths, see: Allen *et al.* (1987 ▶). For the stability of the temperature controller used for the data collection, see: Cosier & Glazer (1986 ▶). For ring conformations, see: Cremer & Pople (1975 ▶).
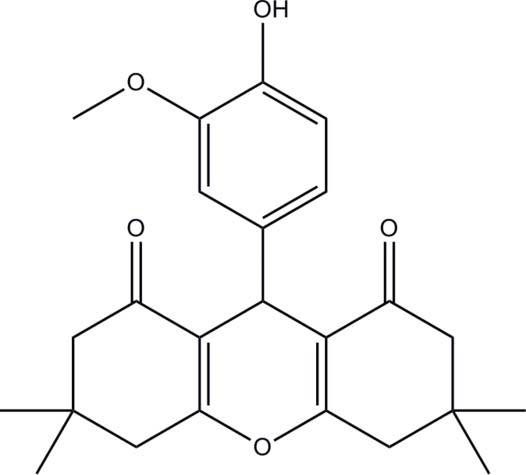


## Experimental

### 

#### Crystal data

C_24_H_28_O_5_
*M* *_r_* = 396.46
Orthorhombic, 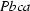

*a* = 11.4861 (10) Å
*b* = 11.8659 (11) Å
*c* = 30.087 (3) Å
*V* = 4100.6 (6) Å^3^
*Z* = 8
Mo *K*α radiation
μ = 0.09 mm^−1^
*T* = 100 K
0.35 × 0.30 × 0.24 mm

#### Data collection

Bruker APEXII DUO CCD area-detector diffractometer
Absorption correction: multi-scan (*SADABS*; Bruker, 2009 ▶) *T*_min_ = 0.970, *T*_max_ = 0.979
26584 measured reflections
5972 independent reflections
4634 reflections with *I* > 2σ(*I*)
*R*_int_ = 0.046

#### Refinement

*R*[*F* ^2^ > 2σ(*F* ^2^)] = 0.045
*wR*(*F* ^2^) = 0.136
*S* = 1.06
5972 reflections
271 parameters
H atoms treated by a mixture of independent and constrained refinement
Δρ_max_ = 0.57 e Å^−3^
Δρ_min_ = −0.38 e Å^−3^

### 

Data collection: *APEX2* (Bruker, 2009 ▶); cell refinement: *SAINT* (Bruker, 2009 ▶); data reduction: *SAINT*; program(s) used to solve structure: *SHELXTL* (Sheldrick, 2008 ▶); program(s) used to refine structure: *SHELXTL*; molecular graphics: *SHELXTL*; software used to prepare material for publication: *SHELXTL* and *PLATON* (Spek, 2009 ▶).

## Supplementary Material

Crystal structure: contains datablocks global, I. DOI: 10.1107/S1600536810020258/rz2454sup1.cif

Structure factors: contains datablocks I. DOI: 10.1107/S1600536810020258/rz2454Isup2.hkl

Additional supplementary materials:  crystallographic information; 3D view; checkCIF report

## Figures and Tables

**Table 1:** Hydrogen-bond geometry (Å, °)

*D*—H⋯*A*	*D*—H	H⋯*A*	*D*⋯*A*	*D*—H⋯*A*
O5—H14*O*⋯O3^i^	0.86 (2)	1.95 (2)	2.7319 (13)	151 (2)
C2—H2*A*⋯O2^ii^	0.97	2.55	3.5003 (16)	165
C20—H20*C*⋯O2^iii^	0.96	2.58	3.4646 (17)	154

Symmetry codes: (i) 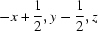; (ii) 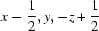; (iii) 
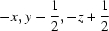
.

**Fig. 1.**
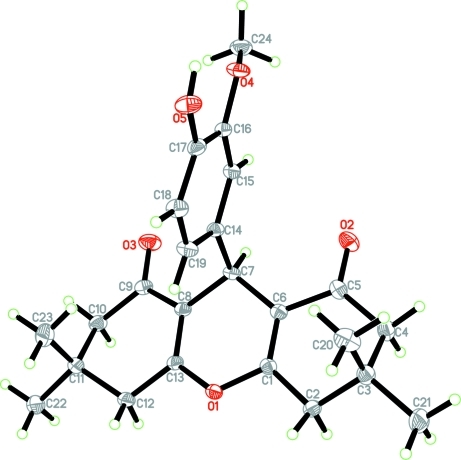
The molecular structure of the title compound, showing 50% probability displacement ellipsoids for non-H atoms and the atom-numbering scheme.

**Fig. 2.**
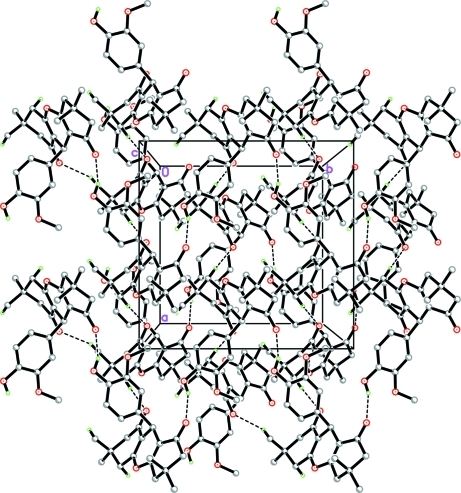
The crystal structure of the title compound viewed along the c axis. H atoms not involved in intermolecular interactions (dashed lines) have been omitted for clarity.

## supplementary crystallographic information

### Comment

Xanthene derivatives especially benzoxanthenes are well-known in organic synthesis due to their biologically active properties such as anti-inflammatory (Hafez *et al.*, 2008), antimicrobial (Ashry *et al.*, 2006), antiviral (Sill & Sweet, 1977) activities and as well as being used in photodynamic therapy (Ion, 1997). Molecules based on the 9,9-dimethylxanthene moiety have inhibitory activity towards trypanothione reductase (TryR) (Chibale *et al.*, 2003). In view of its importance in this field, the crystal structure of the title compound was determined and the results are presented here.

The bond lengths (Allen *et al.*, 1987) and angles in the title compound (Fig. 1) are within normal ranges. The two cyclohexene rings, C1—C6 and C8—C13, adopt an envelope conformation, and the plane through the coplanar atoms makes dihedral angles of 82.86 (6)° and 77.90 (6)°, respectively, with the benzene ring (C14—C19). The puckering parameters (Cremer & Pople, 1975) are Q = 0.4872 (14) Å, Θ = 125.01 (16)° and φ = 307.7 (2)° for the C1—C6 ring, Q = 0.4698 (14) Å, Θ = 56.48 (17)° and φ = 172.6 (2) ° for the C8—C13 ring. The two cyclohexene rings make a dihedral angle of 5.33 (6)° between their least-squares planes. The pyran ring (O1/C1/C6—C8/C13) adopts a flattened boat conformation with atoms C7 and O1 deviating by 0.150 (1) and 0.111 (1) Å, respectively, from the base of the boat.

In the crystal packing (Fig. 2), the molecules are linked into two-dimensional networks parallel to the *ab* plane *via* O5–H14O···O3, C2–H2A···O2 and C20–H20C···O2 interactions (Table 1).

### Experimental

The synthesis of the title compound was performed according to the procedure described in the literature (Venkatesan *et al.*, 2008). A mixture of vanilin (90 mg, 0.59 mmol), dimedone (160 mg, 1.14 mmol), *p*-toluenesulfonic acid (2 mg) in MeOH (4 ml) and water (2 ml) was heated to 50 °C in N_2_ atmosphere for about 20 min. Good quality crystals suitable for characterisation by X-ray crystallography were obtained by recrystallisation from hot methanol.

### Refinement

Atom H14O was located in a difference Fourier map and allowed to refine freely. All other hydrogen atoms were positioned geometrically and refined using a riding model, with C—H = 0.93–0.97 Å and U_iso_(H) = 1.2 or 1.5 U_eq_(C). A rotating-group model was applied for the methyl groups.

### Crystal data

**Table:**

C_24_H_28_O_5_	*F*(000) = 1696
*M**_r_* = 396.46	*D*_x_ = 1.284 Mg m^−^^3^
Orthorhombic, *P**b**c**a*	Mo *K*α radiation, λ = 0.71073 Å
Hall symbol: -P 2ac 2ab	Cell parameters from 5544 reflections
*a* = 11.4861 (10) Å	θ = 2.2–31.4°
*b* = 11.8659 (11) Å	µ = 0.09 mm^−^^1^
*c* = 30.087 (3) Å	*T* = 100 K
*V* = 4100.6 (6) Å^3^	Block, colourless
*Z* = 8	0.35 × 0.30 × 0.24 mm

### Data collection

**Table:**

Bruker APEXII DUO CCD area-detector diffractometer	5972 independent reflections
Radiation source: fine-focus sealed tube	4634 reflections with *I* > 2σ(*I*)
graphite	*R*_int_ = 0.046
φ and ω scans	θ_max_ = 30.0°, θ_min_ = 2.2°
Absorption correction: multi-scan (*SADABS*; Bruker, 2009)	*h* = −16→12
*T*_min_ = 0.970, *T*_max_ = 0.979	*k* = −16→16
26584 measured reflections	*l* = −20→42

### Refinement

**Table:**

Refinement on *F*^2^	Primary atom site location: structure-invariant direct methods
Least-squares matrix: full	Secondary atom site location: difference Fourier map
*R*[*F*^2^ > 2σ(*F*^2^)] = 0.045	Hydrogen site location: inferred from neighbouring sites
*wR*(*F*^2^) = 0.136	H atoms treated by a mixture of independent and constrained refinement
*S* = 1.06	*w* = 1/[σ^2^(*F*_o_^2^) + (0.0729*P*)^2^ + 0.8139*P*] where *P* = (*F*_o_^2^ + 2*F*_c_^2^)/3
5972 reflections	(Δ/σ)_max_ = 0.001
271 parameters	Δρ_max_ = 0.57 e Å^−^^3^
0 restraints	Δρ_min_ = −0.38 e Å^−^^3^

### Special details

**Table:**

Experimental. The crystal was placed in the cold stream of an Oxford Cyrosystems Cobra open-flow nitrogen cryostat (Cosier & Glazer, 1986) operating at 100.0 (1) K.
Geometry. All esds (except the esd in the dihedral angle between two l.s. planes) are estimated using the full covariance matrix. The cell esds are taken into account individually in the estimation of esds in distances, angles and torsion angles; correlations between esds in cell parameters are only used when they are defined by crystal symmetry. An approximate (isotropic) treatment of cell esds is used for estimating esds involving l.s. planes.
Refinement. Refinement of *F*^2^ against ALL reflections. The weighted *R*-factor wR and goodness of fit *S* are based on *F*^2^, conventional *R*-factors *R* are based on *F*, with *F* set to zero for negative *F*^2^. The threshold expression of *F*^2^ > σ(*F*^2^) is used only for calculating *R*-factors(gt) etc. and is not relevant to the choice of reflections for refinement. *R*-factors based on *F*^2^ are statistically about twice as large as those based on *F*, and *R*- factors based on ALL data will be even larger.

### Fractional atomic coordinates and isotropic or equivalent isotropic displacement parameters (Å^2^)

**Table:**

	*x*	*y*	*z*	*U*_iso_*/*U*_eq_	
O1	−0.28861 (7)	0.40281 (8)	0.37337 (3)	0.01617 (19)	
O2	0.01386 (8)	0.44676 (8)	0.26832 (3)	0.0228 (2)	
O3	−0.01627 (8)	0.68315 (9)	0.41472 (4)	0.0257 (2)	
O4	0.35090 (8)	0.35573 (8)	0.36534 (3)	0.0207 (2)	
O5	0.27890 (8)	0.17322 (8)	0.41056 (4)	0.0225 (2)	
C1	−0.23302 (10)	0.38185 (10)	0.33355 (4)	0.0146 (2)	
C2	−0.30172 (10)	0.30335 (11)	0.30502 (4)	0.0170 (2)	
H2A	−0.3616	0.3454	0.2895	0.020*	
H2B	−0.3398	0.2477	0.3236	0.020*	
C3	−0.22358 (11)	0.24318 (11)	0.27099 (4)	0.0190 (3)	
C4	−0.15068 (12)	0.33410 (12)	0.24770 (5)	0.0215 (3)	
H4A	−0.0983	0.2979	0.2268	0.026*	
H4B	−0.2023	0.3826	0.2308	0.026*	
C5	−0.08000 (11)	0.40592 (10)	0.27917 (4)	0.0171 (2)	
C6	−0.12984 (10)	0.42841 (10)	0.32340 (4)	0.0142 (2)	
C7	−0.05937 (10)	0.49443 (10)	0.35702 (4)	0.0142 (2)	
H7A	−0.0228	0.5590	0.3423	0.017*	
C8	−0.14118 (10)	0.53630 (10)	0.39271 (4)	0.0143 (2)	
C9	−0.10776 (11)	0.63245 (11)	0.42079 (4)	0.0172 (2)	
C10	−0.19236 (11)	0.67093 (11)	0.45628 (5)	0.0195 (3)	
H10A	−0.2428	0.7283	0.4438	0.023*	
H10B	−0.1487	0.7054	0.4803	0.023*	
C11	−0.26798 (11)	0.57635 (11)	0.47544 (4)	0.0185 (3)	
C12	−0.32808 (11)	0.51647 (11)	0.43638 (4)	0.0182 (3)	
H12A	−0.3646	0.4479	0.4471	0.022*	
H12B	−0.3888	0.5649	0.4247	0.022*	
C13	−0.24524 (10)	0.48756 (10)	0.39991 (4)	0.0145 (2)	
C14	0.03504 (10)	0.41644 (10)	0.37528 (4)	0.0144 (2)	
C15	0.15186 (10)	0.43068 (10)	0.36324 (4)	0.0150 (2)	
H15A	0.1742	0.4937	0.3470	0.018*	
C16	0.23459 (10)	0.35118 (11)	0.37548 (4)	0.0154 (2)	
C17	0.20159 (11)	0.25532 (10)	0.39955 (4)	0.0163 (2)	
C18	0.08671 (11)	0.24362 (11)	0.41294 (5)	0.0192 (3)	
H18A	0.0648	0.1820	0.4301	0.023*	
C19	0.00419 (11)	0.32338 (11)	0.40088 (5)	0.0186 (3)	
H19A	−0.0726	0.3146	0.4100	0.022*	
C20	−0.14395 (12)	0.15751 (11)	0.29401 (5)	0.0253 (3)	
H20A	−0.1906	0.1025	0.3092	0.038*	
H20B	−0.0951	0.1957	0.3151	0.038*	
H20C	−0.0963	0.1207	0.2722	0.038*	
C21	−0.29993 (13)	0.18345 (13)	0.23661 (5)	0.0291 (3)	
H21A	−0.3498	0.1304	0.2514	0.044*	
H21B	−0.2513	0.1444	0.2157	0.044*	
H21C	−0.3465	0.2381	0.2212	0.044*	
C22	−0.36133 (12)	0.62585 (14)	0.50611 (5)	0.0282 (3)	
H22A	−0.3246	0.6625	0.5308	0.042*	
H22B	−0.4107	0.5664	0.5168	0.042*	
H22C	−0.4072	0.6796	0.4899	0.042*	
C23	−0.19191 (13)	0.49357 (13)	0.50166 (5)	0.0280 (3)	
H23A	−0.1551	0.5324	0.5259	0.042*	
H23B	−0.1334	0.4626	0.4824	0.042*	
H23C	−0.2396	0.4338	0.5131	0.042*	
C24	0.39966 (11)	0.46439 (12)	0.35714 (5)	0.0238 (3)	
H24A	0.4831	0.4594	0.3577	0.036*	
H24B	0.3739	0.5160	0.3797	0.036*	
H24C	0.3748	0.4908	0.3285	0.036*	
H14O	0.3488 (19)	0.1956 (19)	0.4055 (8)	0.047 (6)*	

### Atomic displacement parameters (Å^2^)

**Table:**

	*U*^11^	*U*^22^	*U*^33^	*U*^12^	*U*^13^	*U*^23^
O1	0.0129 (4)	0.0191 (4)	0.0165 (4)	−0.0030 (3)	0.0024 (3)	−0.0032 (3)
O2	0.0224 (5)	0.0237 (5)	0.0223 (5)	−0.0030 (4)	0.0075 (4)	0.0022 (4)
O3	0.0185 (5)	0.0247 (5)	0.0339 (6)	−0.0067 (4)	0.0031 (4)	−0.0047 (4)
O4	0.0105 (4)	0.0246 (5)	0.0272 (5)	0.0030 (3)	0.0023 (3)	0.0053 (4)
O5	0.0165 (5)	0.0195 (5)	0.0315 (5)	0.0044 (4)	−0.0038 (4)	0.0044 (4)
C1	0.0140 (5)	0.0146 (5)	0.0152 (5)	0.0022 (4)	0.0005 (4)	0.0006 (4)
C2	0.0153 (5)	0.0179 (5)	0.0179 (6)	−0.0010 (4)	−0.0009 (4)	−0.0017 (5)
C3	0.0214 (6)	0.0169 (6)	0.0187 (6)	−0.0027 (5)	0.0022 (5)	−0.0026 (5)
C4	0.0263 (7)	0.0216 (6)	0.0166 (6)	−0.0029 (5)	0.0036 (5)	−0.0008 (5)
C5	0.0205 (6)	0.0143 (5)	0.0166 (6)	0.0021 (4)	0.0024 (5)	0.0028 (5)
C6	0.0132 (5)	0.0135 (5)	0.0159 (5)	0.0021 (4)	0.0003 (4)	0.0024 (4)
C7	0.0106 (5)	0.0146 (5)	0.0173 (6)	0.0004 (4)	0.0012 (4)	0.0017 (4)
C8	0.0114 (5)	0.0148 (5)	0.0166 (5)	0.0015 (4)	−0.0001 (4)	0.0015 (4)
C9	0.0144 (5)	0.0166 (5)	0.0206 (6)	0.0004 (4)	−0.0021 (5)	0.0007 (5)
C10	0.0165 (6)	0.0202 (6)	0.0219 (6)	−0.0002 (5)	−0.0008 (5)	−0.0049 (5)
C11	0.0155 (5)	0.0237 (6)	0.0163 (6)	−0.0008 (5)	0.0010 (5)	−0.0023 (5)
C12	0.0127 (5)	0.0229 (6)	0.0191 (6)	−0.0005 (4)	0.0017 (4)	−0.0036 (5)
C13	0.0119 (5)	0.0161 (5)	0.0156 (5)	0.0006 (4)	−0.0011 (4)	−0.0001 (5)
C14	0.0116 (5)	0.0152 (5)	0.0164 (5)	0.0014 (4)	−0.0005 (4)	−0.0004 (4)
C15	0.0129 (5)	0.0156 (5)	0.0164 (5)	−0.0004 (4)	0.0002 (4)	0.0008 (4)
C16	0.0112 (5)	0.0199 (6)	0.0151 (5)	0.0013 (4)	−0.0007 (4)	−0.0016 (5)
C17	0.0141 (5)	0.0177 (6)	0.0171 (6)	0.0026 (4)	−0.0031 (4)	−0.0001 (5)
C18	0.0169 (6)	0.0173 (6)	0.0235 (6)	−0.0005 (4)	−0.0003 (5)	0.0043 (5)
C19	0.0130 (5)	0.0193 (6)	0.0235 (6)	−0.0002 (4)	0.0016 (5)	0.0028 (5)
C20	0.0260 (7)	0.0145 (6)	0.0354 (8)	0.0013 (5)	0.0065 (6)	0.0001 (6)
C21	0.0334 (8)	0.0288 (7)	0.0252 (7)	−0.0090 (6)	0.0034 (6)	−0.0102 (6)
C22	0.0221 (7)	0.0409 (8)	0.0216 (7)	−0.0028 (6)	0.0033 (5)	−0.0115 (6)
C23	0.0301 (7)	0.0339 (8)	0.0200 (7)	0.0016 (6)	−0.0021 (6)	0.0045 (6)
C24	0.0143 (6)	0.0272 (7)	0.0298 (7)	−0.0045 (5)	0.0002 (5)	−0.0047 (6)

### Geometric parameters (Å, °)

**Table:**

O1—C13	1.3772 (15)	C11—C22	1.5317 (18)
O1—C1	1.3802 (14)	C11—C23	1.5333 (19)
O2—C5	1.2263 (15)	C11—C12	1.5370 (18)
O3—C9	1.2246 (15)	C12—C13	1.4922 (17)
O4—C16	1.3714 (15)	C12—H12A	0.9700
O4—C24	1.4272 (17)	C12—H12B	0.9700
O5—C17	1.3591 (15)	C14—C19	1.3922 (18)
O5—H14O	0.86 (2)	C14—C15	1.4001 (16)
C1—C6	1.3427 (17)	C15—C16	1.3886 (17)
C1—C2	1.4924 (17)	C15—H15A	0.9300
C2—C3	1.5372 (18)	C16—C17	1.4007 (18)
C2—H2A	0.9700	C17—C18	1.3867 (18)
C2—H2B	0.9700	C18—C19	1.3877 (18)
C3—C21	1.5301 (19)	C18—H18A	0.9300
C3—C20	1.5329 (19)	C19—H19A	0.9300
C3—C4	1.5350 (18)	C20—H20A	0.9600
C4—C5	1.5106 (19)	C20—H20B	0.9600
C4—H4A	0.9700	C20—H20C	0.9600
C4—H4B	0.9700	C21—H21A	0.9600
C5—C6	1.4730 (17)	C21—H21B	0.9600
C6—C7	1.5140 (17)	C21—H21C	0.9600
C7—C8	1.5108 (17)	C22—H22A	0.9600
C7—C14	1.5278 (16)	C22—H22B	0.9600
C7—H7A	0.9800	C22—H22C	0.9600
C8—C13	1.3454 (16)	C23—H23A	0.9600
C8—C9	1.4706 (17)	C23—H23B	0.9600
C9—C10	1.5143 (18)	C23—H23C	0.9600
C10—C11	1.5317 (18)	C24—H24A	0.9600
C10—H10A	0.9700	C24—H24B	0.9600
C10—H10B	0.9700	C24—H24C	0.9600
			
C13—O1—C1	117.88 (9)	C11—C12—H12A	109.1
C16—O4—C24	117.15 (10)	C13—C12—H12B	109.1
C17—O5—H14O	110.3 (15)	C11—C12—H12B	109.1
C6—C1—O1	122.11 (11)	H12A—C12—H12B	107.8
C6—C1—C2	126.34 (11)	C8—C13—O1	122.80 (11)
O1—C1—C2	111.55 (10)	C8—C13—C12	125.88 (11)
C1—C2—C3	111.36 (10)	O1—C13—C12	111.32 (10)
C1—C2—H2A	109.4	C19—C14—C15	118.86 (11)
C3—C2—H2A	109.4	C19—C14—C7	119.92 (10)
C1—C2—H2B	109.4	C15—C14—C7	120.94 (11)
C3—C2—H2B	109.4	C16—C15—C14	120.35 (12)
H2A—C2—H2B	108.0	C16—C15—H15A	119.8
C21—C3—C20	109.88 (12)	C14—C15—H15A	119.8
C21—C3—C4	109.24 (11)	O4—C16—C15	125.52 (12)
C20—C3—C4	110.30 (11)	O4—C16—C17	114.24 (11)
C21—C3—C2	109.31 (11)	C15—C16—C17	120.23 (11)
C20—C3—C2	110.83 (11)	O5—C17—C18	118.62 (12)
C4—C3—C2	107.22 (10)	O5—C17—C16	122.09 (11)
C5—C4—C3	113.79 (11)	C18—C17—C16	119.28 (11)
C5—C4—H4A	108.8	C17—C18—C19	120.37 (12)
C3—C4—H4A	108.8	C17—C18—H18A	119.8
C5—C4—H4B	108.8	C19—C18—H18A	119.8
C3—C4—H4B	108.8	C18—C19—C14	120.78 (12)
H4A—C4—H4B	107.7	C18—C19—H19A	119.6
O2—C5—C6	120.71 (12)	C14—C19—H19A	119.6
O2—C5—C4	121.91 (12)	C3—C20—H20A	109.5
C6—C5—C4	117.36 (11)	C3—C20—H20B	109.5
C1—C6—C5	118.29 (11)	H20A—C20—H20B	109.5
C1—C6—C7	122.20 (11)	C3—C20—H20C	109.5
C5—C6—C7	119.31 (10)	H20A—C20—H20C	109.5
C8—C7—C6	108.21 (10)	H20B—C20—H20C	109.5
C8—C7—C14	112.65 (10)	C3—C21—H21A	109.5
C6—C7—C14	107.84 (10)	C3—C21—H21B	109.5
C8—C7—H7A	109.4	H21A—C21—H21B	109.5
C6—C7—H7A	109.4	C3—C21—H21C	109.5
C14—C7—H7A	109.4	H21A—C21—H21C	109.5
C13—C8—C9	118.22 (11)	H21B—C21—H21C	109.5
C13—C8—C7	121.72 (11)	C11—C22—H22A	109.5
C9—C8—C7	120.07 (10)	C11—C22—H22B	109.5
O3—C9—C8	121.30 (12)	H22A—C22—H22B	109.5
O3—C9—C10	120.51 (12)	C11—C22—H22C	109.5
C8—C9—C10	118.14 (11)	H22A—C22—H22C	109.5
C9—C10—C11	114.09 (11)	H22B—C22—H22C	109.5
C9—C10—H10A	108.7	C11—C23—H23A	109.5
C11—C10—H10A	108.7	C11—C23—H23B	109.5
C9—C10—H10B	108.7	H23A—C23—H23B	109.5
C11—C10—H10B	108.7	C11—C23—H23C	109.5
H10A—C10—H10B	107.6	H23A—C23—H23C	109.5
C22—C11—C10	110.04 (11)	H23B—C23—H23C	109.5
C22—C11—C23	109.54 (12)	O4—C24—H24A	109.5
C10—C11—C23	109.87 (11)	O4—C24—H24B	109.5
C22—C11—C12	108.88 (10)	H24A—C24—H24B	109.5
C10—C11—C12	107.80 (11)	O4—C24—H24C	109.5
C23—C11—C12	110.68 (11)	H24A—C24—H24C	109.5
C13—C12—C11	112.46 (10)	H24B—C24—H24C	109.5
C13—C12—H12A	109.1		
			
C13—O1—C1—C6	10.47 (17)	C9—C10—C11—C22	−172.25 (11)
C13—O1—C1—C2	−169.61 (10)	C9—C10—C11—C23	67.06 (14)
C6—C1—C2—C3	22.80 (18)	C9—C10—C11—C12	−53.64 (14)
O1—C1—C2—C3	−157.11 (10)	C22—C11—C12—C13	167.54 (12)
C1—C2—C3—C21	−168.27 (11)	C10—C11—C12—C13	48.19 (14)
C1—C2—C3—C20	70.47 (13)	C23—C11—C12—C13	−71.99 (14)
C1—C2—C3—C4	−49.96 (14)	C9—C8—C13—O1	174.91 (11)
C21—C3—C4—C5	174.64 (12)	C7—C8—C13—O1	−5.31 (18)
C20—C3—C4—C5	−64.49 (15)	C9—C8—C13—C12	−4.82 (19)
C2—C3—C4—C5	56.29 (14)	C7—C8—C13—C12	174.95 (12)
C3—C4—C5—O2	148.98 (12)	C1—O1—C13—C8	−12.05 (17)
C3—C4—C5—C6	−32.87 (16)	C1—O1—C13—C12	167.72 (10)
O1—C1—C6—C5	−176.84 (10)	C11—C12—C13—C8	−21.12 (18)
C2—C1—C6—C5	3.25 (18)	C11—C12—C13—O1	159.11 (10)
O1—C1—C6—C7	8.37 (18)	C8—C7—C14—C19	52.88 (15)
C2—C1—C6—C7	−171.54 (11)	C6—C7—C14—C19	−66.46 (15)
O2—C5—C6—C1	179.82 (11)	C8—C7—C14—C15	−133.24 (12)
C4—C5—C6—C1	1.65 (17)	C6—C7—C14—C15	107.43 (13)
O2—C5—C6—C7	−5.23 (17)	C19—C14—C15—C16	2.08 (19)
C4—C5—C6—C7	176.59 (11)	C7—C14—C15—C16	−171.87 (11)
C1—C6—C7—C8	−22.67 (15)	C24—O4—C16—C15	25.08 (18)
C5—C6—C7—C8	162.59 (10)	C24—O4—C16—C17	−156.10 (12)
C1—C6—C7—C14	99.45 (13)	C14—C15—C16—O4	179.50 (12)
C5—C6—C7—C14	−75.29 (13)	C14—C15—C16—C17	0.74 (19)
C6—C7—C8—C13	21.08 (15)	O4—C16—C17—O5	−1.71 (18)
C14—C7—C8—C13	−98.04 (13)	C15—C16—C17—O5	177.18 (12)
C6—C7—C8—C9	−159.15 (11)	O4—C16—C17—C18	177.85 (12)
C14—C7—C8—C9	81.73 (14)	C15—C16—C17—C18	−3.26 (19)
C13—C8—C9—O3	−177.09 (12)	O5—C17—C18—C19	−177.48 (12)
C7—C8—C9—O3	3.13 (18)	C16—C17—C18—C19	3.0 (2)
C13—C8—C9—C10	0.12 (17)	C17—C18—C19—C14	−0.1 (2)
C7—C8—C9—C10	−179.65 (11)	C15—C14—C19—C18	−2.4 (2)
O3—C9—C10—C11	−152.16 (12)	C7—C14—C19—C18	171.61 (12)
C8—C9—C10—C11	30.60 (16)		

### Hydrogen-bond geometry (Å, °)

**Table:**

*D*—H···*A*	*D*—H	H···*A*	*D*···*A*	*D*—H···*A*
O5—H14O···O3^i^	0.86 (2)	1.95 (2)	2.7319 (13)	151 (2)
C2—H2A···O2^ii^	0.97	2.55	3.5003 (16)	165
C20—H20C···O2^iii^	0.96	2.58	3.4646 (17)	154

Symmetry codes: (i) −*x*+1/2, *y*−1/2, *z*; (ii) *x*−1/2, *y*, −*z*+1/2; (iii) −*x*, *y*−1/2, −*z*+1/2.

## References

[bb1] Allen, F. H., Kennard, O., Watson, D. G., Brammer, L., Orpen, A. G. & Taylor, R. (1987). *J. Chem. Soc. Perkin Trans. 2*, pp. S1–19.

[bb2] Ashry, E. S. H. E., Awad, L. F., Ibrahim, E. S. I. & Bdeewy, O. K. (2006). *Ar*kivoc, **2**, 178–186.

[bb3] Bruker (2009). *APEX2*, *SAINT* and *SADABS* Bruker AXS Inc., Madison, Wisconsin, USA.

[bb4] Chibale, K., Visser, M., Schalkwyk, D., Smith, P. J., Saravanamuthu, A. & Fairlamb, A. H. (2003). *Tetrahedron*, **59**, 2289–2296.

[bb5] Cosier, J. & Glazer, A. M. (1986). *J. Appl. Cryst.* **19**, 105–107.

[bb6] Cremer, D. & Pople, J. A. (1975). *J. Am. Chem. Soc.* **97**, 1354–1358.

[bb7] Hafez, H. N., Hegab, M. I., Ahmed-Farag, I. S. & El-Gazzar, A. B. A. (2008). *Bioorg. Med. Chem. Lett.* **18**, 4538–4543. 10.1016/j.bmcl.2008.07.042 18667305

[bb8] Ion, R. M. (1997). *Prog. Catal.* **2**, 55–76.

[bb9] Sheldrick, G. M. (2008). *Acta Cryst.* A**64**, 112–122. 10.1107/S0108767307043930 18156677

[bb10] Sill, A. D. & Sweet, F. W. (1977). US Patent No. 4008240.

[bb11] Spek, A. L. (2009). *Acta Cryst.* D**65**, 148–155. 10.1107/S090744490804362X PMC2631630 19171970

[bb12] Venkatesan, K., Pujari, S. S., Lahoti, R. J. & Srinivasan, K. V. (2008). *Ultrason. Sonochem.* **15**, 548–553. 10.1016/j.ultsonch.2007.06.001 17658286

